# Regulation of *CDX4* gene transcription by HoxA9, HoxA10, the Mll-Ell oncogene and Shp2 during leukemogenesis

**DOI:** 10.1038/oncsis.2014.49

**Published:** 2014-12-22

**Authors:** L Bei, C Shah, H Wang, W Huang, L C Platanias, E A Eklund

**Affiliations:** 1 Department of Medicine, The Feinberg School of Medicine, Northwestern University, Chicago, IL, USA; 2 The Robert H. Lurie Comprehensive Cancer Center, Northwestern University, Chicago, IL, USA; 3 Jesse Brown VA Medical Center, Chicago, IL, USA

## Abstract

Cdx and Hox proteins are homeodomain transcription factors that regulate hematopoiesis. Transcription of the *HOX* and *CDX* genes decreases during normal myelopoiesis, but is aberrantly sustained in leukemias with translocation or partial tandem duplication of the *MLL1* gene. Cdx4 activates transcription of the *HOXA9* and *HOXA10* genes, and HoxA10 activates *CDX4* transcription. The events that break this feedback loop, permitting a decreased Cdx4 expression during normal myelopoiesis, were previously undefined. In the current study, we find that HoxA9 represses *CDX4* transcription in differentiating myeloid cells, antagonizing activation by HoxA10. We determine that tyrosine phosphorylation of HoxA10 impairs transcriptional activation of *CDX4*, but tyrosine phosphorylation of HoxA9 facilitates repression of this gene. As HoxA9 and HoxA10 are phosphorylated during myelopoiesis, this provides a mechanism for differentiation stage-specific Cdx4 expression. HoxA9 and HoxA10 are increased in cells expressing Mll-Ell, a leukemia-associated *MLL1* fusion protein. We find that Mll-Ell induces a HoxA10-dependent increase in Cdx4 expression in myeloid progenitor cells. However, Cdx4 decreases in a HoxA9-dependent manner on exposure of Mll-Ell-expressing cells to differentiating cytokines. Leukemia-associated, constitutively active mutants of Shp2 block cytokine-induced tyrosine phosphorylation of HoxA9 and HoxA10. In comparison with myeloid progenitor cells that are expressing Mll-Ell alone, we find increased *CDX4* transcription and Cdx4 expression in cells co-expressing Mll-Ell plus constitutively active Shp2. Increased Cdx4 expression is sustained on exposure of these cells to differentiating cytokines. Our results identify a mechanism for increased and sustained *CDX4* transcription in leukemias co-overexpressing HoxA9 and HoxA10 in combination with constitutive activation of Shp2. This is clinically relevant, because *MLL1* translocations and constitutive Shp2 activation co-exist in human myeloid leukemias.

## Introduction

*HOX* genes are found in four groups on four chromosomes and encode a set of highly conserved homeodomain (HD) transcription factors.^1^ During hematopoiesis, Hox1–4 are maximally expressed in hematopoietic stem cells, whereas Hox7–11 are expressed in committed progenitors.^2^ *HOX* transcription decreases during normal myelopoiesis, but a subset of poor prognosis, acute leukemias are characterized by increased and sustained transcription of a group of *HOX* genes (HoxB3, B4, A7–11).^3, 4, 5^ This includes leukemias with chromosomal translocations of the *MLL1* gene (referred to as 11q23-leukemia).^6, 7, 8, 9^ The Mixed Lineage Leukemia 1 (Mll1) protein activates *HOX* transcription in hematopoietic stem and progenitor cells, but ubiquitination-mediated degradation of Mll1 decreases this effect during normal hematopoiesis.^6, 7, 8, 9, 10^ Fusion proteins generated by *MLL1* gene translocations lack ubiquitinated domains, resulting in sustained *HOX* transcription.^10^ Engineered loss of Mll1 in mice impairs *HOX* transcription and results in hematopoietic defects.^11^ Conversely, mice that are transplanted with bone marrow expressing an Mll-fusion protein, or overexpressing HoxA9 or HoxA10, develop a myeloproliferative neoplasm that progresses to acute myeloid leukemia (AML) over time.^12, 13, 14, 15^

Cdx proteins are HD transcription factors that also regulate *HOX* transcription. Loss of Cdx4 in mice decreases the expression of HoxA9 and HoxA10 and impairs hematopoiesis.^16, 17^ Engineered overexpression of Cdx4 is leukemogenic in mice, and rescues Hox expression in *MLL1*−/− bone marrow.^16, 17^ *HOXA9* and *HOXA10* are Cdx4 target genes and *CDX4* is a sHoxA10 target gene.^18, 19^ This defines a positive feedback mechanism whereby Cdx4 enhances expression of HoxA9 and HoxA10, and HoxA10 enhances Cdx4 expression. A decrease in *CDX4* transcription during myelopoiesis implies the existence of a mechanism that disrupts this feedback. In the current study, we hypothesize that HoxA9 represses *CDX4* in differentiating cells, antagonizing HoxA10.

HoxA9 and HoxA10 co-regulate *CYBB* and *NCF2*; genes that encode components of the phagocyte NADPH-oxidase.^20, 21, 22, 23, 24, 25, 26^ HoxA10 binds to and represses homologous *CYBB* and *NCF2* *cis* elements in myeloid progenitor cells. Differentiating cytokines induce the phosphorylation of tyrosine residues in the HoxA10-HD, decreasing the binding affinity for these genes.^20, 22, 23, 24^ HoxA9 binds the same *CYBB* and *NCF2* *cis* elements and activates transcription during myelopoiesis in a manner that is facilitated by phosphorylation of conserved HD tyrosine residues in HoxA9.^21^ In myeloid progenitor cells, HoxA9 and HoxA10 are maintained in a nonphosphorylated state by Shp2.^24^ However, leukemia-associated, constitutively active mutants of Shp2 dephosphorylate HoxA9 and HoxA10 throughout myelopoiesis.^24^ Mice transplanted with bone marrow that is co-overexpressing HoxA10+activated Shp2 (E76K) develop AML without the lag time that is required with HoxA10 overexpression alone.^12^ Sustained *CYBB* and *NCF2* repression contributes to phenotypic differentiation block in these mice.^12^

In the current study, we find that HoxA9 and HoxA10 co-regulate *CDX4* via novel mechanisms, activation by HoxA10 in progenitors and repression by HoxA9 during myelopoiesis. Also, HoxA9 and HoxA10 interact with different *CDX4* *cis* elements, unlike previously described common target genes. Mll-fusion proteins increase the expression of both HoxA9 and HoxA10, and we find that Mll-Ell enhances *CDX4* transcription in progenitor cells, but decreases transcription in cells exposed to differentiating cytokines. However, Cdx4 expression is increased and sustained in cytokine-stimulated cells co-expressing Mll-Ell plus E76K-Shp2. This is clinically relevant because Shp2 activation coexists with *MLL1* translocations in human AML.^27^ Our studies describe a mechanism for increased and sustained Cdx4 expression in Hox-overexpressing leukemias.

## Results

### HoxA9 represses CDX4 transcription

**Figure 1:**
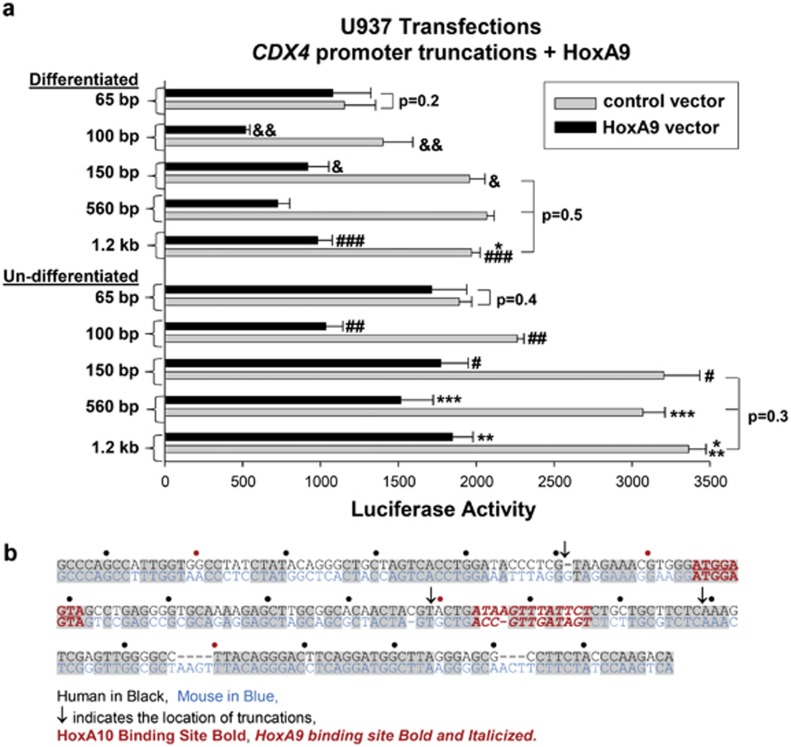
HoxA9 represses *CDX4* transcription. (**a**) HoxA9 decreases *CDX4* promoter activity. U937 myeloid cells were transfected with a series of *CDX4* promoter–reporter constructs and a vector to overexpress HoxA9 or control expression vector. The reporter activity was determined with or without differentiation with retinoic acid/dimethyl formamide. A statistically significant difference in reporter activity with versus without differentiation is indicated by *. Statistically significant differences in reporter activity with HoxA9 overexpression versus control vector are indicated by **, ***, #, ##, ###, & or &&. Differences with *P*<0.01 are considered statistically significant. Some nonstatistically significant differences are also indicated. (**b**) The *CDX4* promoter includes multiple Hox DNA-binding consensus sequences. The sequence of the proximal human *CDX4* 5′ flank (from the transcription start site) is depicted in black and the murine sequence in blue. Conserved sequences are indicated in gray, the distal HoxA10-binding *cis* element is indicated in red, the proximal HoxA9-binding *cis* element is indicated in red and italicized and the site of truncations used in reporter assays are indicated by arrows.

The *CDX4* promoter includes other Hox-binding consensus sequences in addition to the previously identified, HoxA10-binding *cis* element at −139 to −150 bp (relative to the transcription start site).^19^ To identify HoxA9-regulated *cis* elements, we employed a series of promoter truncations designed around these consensus sequences.^19^ *CDX4* promoter fragments were subcloned into a reporter vector and co-transfected into U937 myeloid cells with a vector to overexpress HoxA9 or control expression vector. To investigate the dynamics of *CDX4* transcription during granulopoiesis, transfectants were analyzed with or without differentiation with retinoic acid/dimethyl formamide (RA/DMF).^28, 29^ We found that the overexpression of HoxA9 significantly decreased the activity of constructs with 1.2 kb, 560, 150 and 100 bp of *CDX4* promoter in transfectants relative to control transfectants (*P*<0.01, *n*=6 for HoxA9 versus control expression vector; Figure 1a). This effect of HoxA9 was not observed with the 65 bp *CDX4* promoter construct (*P*>0.5, *n*=6), suggesting that HoxA9 influences a cis element between −65 and −100 bp rather than the more distal HoxA10-binding *cis* element (Figure 1b).

**Figure 2:**
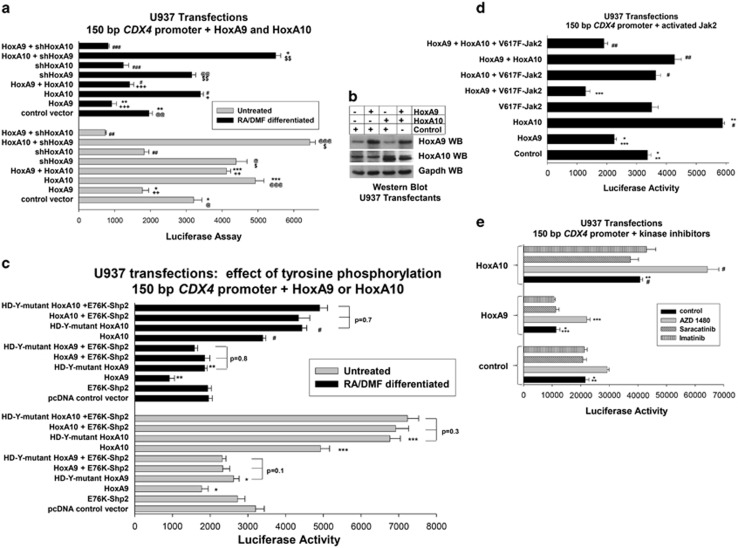
HoxA9 and HoxA10 are antagonists for *CDX4* promoter activity during myelopoiesis. (**a**) HoxA9 antagonizes the effect of HoxA10 on the *CDX4* promoter. U937 cells were transfected with a reporter construct with 150 bp of *CDX4* promoter and combinations of vectors to overexpress or knockdown HoxA9 or HoxA10. Reporter activity was determined with or without differentiation. Statistically significant differences in reporter activity are indicated by *, **, ***, #, ##, ### for with versus without overexpressed HoxA9; @, @@, @@@ or + for with versus without HoxA9 shRNA; ++, +++, $ or $$ for with versus without HoxA10 overexpression. (**b**) HoxA9 and HoxA10 protein are equivalently overexpressed in U937 transfectants. U937 cells were transfected with vectors to overexpress HoxA9, HoxA10, HoxA9+HoxA10 or control expression vector. Western blots were serially probed with antibodies to HoxA9, HoxA10 and Gapdh (as a loading control). (**c**) Tyrosine phosphorylation decreases the repression of the *CDX4* promoter by HoxA9 and increases activation by HoxA10. U937 cells were transfected with a reporter construct with 150 bp of *CDX4* promoter and vectors to overexpress tyrosine mutant HoxA9 (HD-Y-mutant HoxA9) or tyrosine mutant HoxA10 (HD-Y-mutant HoxA10) or vectors to overexpress wild-type or HD-Y-mutant HoxA9 or HoxA10 plus constitutively active Shp2 (E76K). Reporter activity was determined with or without differentiation. Statistically significant differences in reporter activity with wild-type versus tyrosine mutant protein are indicated by *, **, *** or #. Some differences that are not statistically significant (*P*>0.1) are indicated. (**d**) Constitutively active Jak2 increases repression of the *CDX4* promoter by HoxA9 and decreases activation by HoxA10. U937 cells were transfected with a reporter construct with 150 bp of *CDX4* promoter and a vector to express constitutively active Jak2 (V617F) with or without vectors to overexpress HoxA9, HoxA10 or both (or control vector). Statistically significant differences in reporter activity with HoxA9 or HoxA10 versus control vector are indicated by * or **. Statistically significant differences with versus without V617F-Jak2 are indicated by ***, # or ##. (**e**) Inhibition of Jak2 blocks repression of the *CDX4* promoter by HoxA9 but increases activation by HoxA10 in differentiating myeloid cells, but inhibition of Src or Abl tyrosine kinase do not have a similar effect. U937 cells were transfected with a reporter construct with 150 bp of *CDX4* promoter and vectors to overexpress HoxA9 or HoxA10 (or control vector). Cells were differentiated with retinoic acid/dimethyl formamide (RA/DMF) and some were treated with AZD 1480 (Jak2 inhibitor), Sarcatinib (Src inhibitor) or Imatinib (Ableson kinase inhibitor). Statistically significant differences in reporter activity with HoxA9 or HoxA10 overexpression versus control vector are indicated by * and **. Statistically significant differences with versus without Jak2-inhibition are indicated by *** or #.

We investigated the combined effects of HoxA9 and HoxA10 by co-transfecting U937 cells with the 150-bp *CDX4* reporter construct (the shortest one influenced by both Hox proteins) and vectors to overexpress these proteins (or control vector). The activity of the 150-bp construct in undifferentiated U937 cells co-overexpressing HoxA9+HoxA10 was significantly greater than transfectants with control expression vector (*P*<0.01, *n*=6), but significantly less than transfectants overexpressing HoxA10 alone (*P*<0.02, *n*=6; Figure 2a). In differentiated transfectants, the activity of the 150-bp construct was significantly less in cells co-overexpressing HoxA9+HoxA10 in comparison with control vector transfectants (*P*<0.001, *n*=6), but repression was more efficient with HoxA9 overexpression alone versus co-overexpression of HoxA9+HoxA10 (*P*<0.01, *n*=6; Figure 2a). HoxA9 and HoxA10 were equivalently overexpressed in these cells (Figure 2b).

To further investigate the relative effects of HoxA9 and HoxA10, we co-transfected U937 cells with the 150-bp *CDX4* promoter construct and vectors to express HoxA9- or HoxA10-specific short hairpin RNA (shRNAs; or scrambled control shRNAs) or to overexpress one Hox protein and knockdown the other. Activity of the 150-bp *CDX4* promoter construct was significantly greater in transfectants with HoxA9-specific shRNA relative to transfectants with control shRNA and knockdown of HoxA9 also augmented activation by overexpressed HoxA10 (*P*<0.01, *n*=6 for both comparisons; Figure 2a). HoxA10 knockdown significantly decreased *CDX4* promoter activity relative to transfectants with control shRNA and augmented repression by HoxA9 (*P*<0.001, *n*=6 for both comparisons; Figure 2a). The effects of HoxA9 knockdown were significantly greater in differentiated versus undifferentiated transfectants (*P*=0.003, *n*=6), and effects of HoxA10 knockdown were significantly greater in undifferentiated transfectants versus differentiated transfectants (*P*=0.01, *n*=6).

We investigated the contribution of tyrosine phosphorylation of HoxA9 or HoxA10 to *CDX4* regulation by expressing forms of these proteins with conserved HD tyrosine residues mutated to phenylalanine (Y212F/Y225F-HoxA9 or Y326F/Y343F-HoxA10). These mutations influence the transcription of the *CYBB* and *NCF2* genes, but not Hox protein stability.^21, 23, 24^ We co-transfected U937 cells with the 150-bp *CDX4* reporter construct and vectors to overexpress HD-Y-mutant HoxA9 or HD-Y-mutant HoxA10. Repression of *CDX4* by HD-Y-mutant HoxA9 was significantly less efficient than wild-type HoxA9 (*P*<0.0001, *n*=6; Figure 2c). Conversely, HD-Y-mutant HoxA10 was significantly more efficient in activating the *CDX4* promoter than wild-type HoxA10 (*P*<0.001, *n*=6; Figure 2c). As an additional method to prevent HoxA9 and HoxA10 phosphorylation, some cells were co-transfected with vectors to express constitutively active Shp2 (E76K-Shp2).^12, 24^ E76K-Shp2 significantly decreased the efficiency of *CDX4* repression by overexpressed HoxA9 (*P*<0.01, *n*=4 for comparison with HoxA9 alone), but E76K-Shp2 significantly increased *CDX4* activation by overexpressed HoxA10 (*P*<0.001, *n*=4 for comparison with HoxA10 alone; Figure 2c).

In these studies, we titrated the expression of E76K-Shp2 so that it had an minimal effect on reporter activity in the absence of overexpressed HoxA9 or HoxA10. As an additional control experiment, we investigated the impact of this level of constitutive Shp2 activity on overexpressed, HD-Y-mutant HoxA9 or HoxA10. We found that the activity of the 150 bp *CDX4* promoter construct was not significantly different in transfectants co-overexpressing HD-Y-mutant HoxA9+E76K-Shp2 versus HD-Y-mutant HoxA9 (*P*⩾0.1, *n*=4), or in transfectants co-overexpressing HD-Y-mutant HoxA10+E76K-Shp2 versus HD-Y-mutant HoxA10 (*P*⩾0.3, *n*=4; Figure 2c).

As we previously determined that various cytokines induce tyrosine phosphorylation of HoxA9 and HoxA10 in a Jak2-dependent manner, we investigated the effect of Jak2 on *CDX4* promoter activity.^30^ For these studies, we co-transfected U937 cells with the 150-bp *CDX4* promoter construct, a vector to express constitutively active Jak2 (V617F), and vectors to overexpress HoxA9 or HoxA10 (or relevant control vectors). We found less activation of the *CDX4* promoter in transfectants overexpressing HoxA10+V617F-Jak2 in comparison with HoxA10 alone (*P*<0.001, *n*=3; Figure 2d). Conversely, we found significantly greater repression of the *CDX4* promoter in transfectants overexpressing HoxA9+V617F- Jak2 versus HoxA9 alone (*P*<0.01, *n*=3; Figure 2d). Similar to differentiation, the addition of activated Jak2 to transfectants co-overexpressing HoxA9+HoxA10 switched the net effect from activation to repression (Figure 2d).

We also investigated the effect on *CDX4* promoter activity of inhibiting Jak2 or other tyrosine kinases involved in myelopoiesis. In these studies, we co-transfected U937 cells with the 150-bp *CDX4* promoter construct and a vector to overexpress HoxA9 or HoxA10 (or control vector). Cells were differentiated with retinoic acid/dimethyl formamide and analyzed for reporter activity after treatment with a Jak2 inhibitor (AZD 1480), a Src inhibitor (Saracatinib) or an Abl inhibitor (imatinib).^31, 32, 33^ We found that treatment with the Jak2 inhibitor blocked HoxA9-induced repression of the 150-bp *CDX4* promoter (*P*<0.001, *n*=6 with versus without AZD 1480), but increased activation of the *CDX4* promoter by HoxA10 (*P*<0.001, *n*=6 with versus without AZD 1480; Figure 2e). In contrast, inhibition of Src or Abl did not influence activity of the *CDX4* promoter, with or without overexpressed HoxA9 or HoxA10 (Figure 2e).

Activity of the empty reporter vector was not influenced by differentiation or manipulation of HoxA9 or HoxA10 and was subtracted as background. We previously demonstrated that these shRNA vectors are specific for HoxA9 or HoxA10 and decrease the endogenous proteins by ~70%.^28, 34^

### HoxA9 and HoxA10 influence adjacent CDX4 *cis* elements

**Figure 3:**
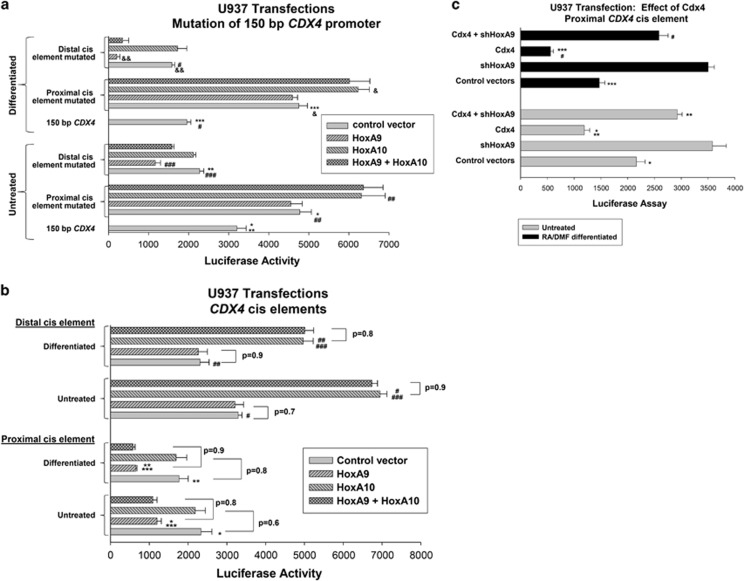
HoxA9 and HoxA10 influence distinct *CDX4* promoter *cis* elements. (**a**) The HoxA9-induced repression requires the proximal *CDX4* promoter *cis* element and activation by HoxA10 the distal *cis* element. U937 cells were transfected with a reporter construct with 150 bp of *CDX4* promoter with mutation in the Hox-binding consensus between −80 and −93 bp, the Hox-binding consensus between −141 and −148 or neither. Cells were co-transfected with vectors to overexpress HoxA9, HoxA10 or both (or control vector) and reporter assays performed with or without differentiation. Statistically significant differences in reporter activity are indicated by * and *** for mutation of the HoxA10-binding *cis* element; ** or # for mutation of the HoxA9 influenced *cis* element; ## or & for the overexpression of HoxA10 versus control expression vector; ### or && for the overexpression of HoxA9 versus control expression vector. Differences of *P*<0.01 are considered statistically significant. Some nonstatistically significant differences are also indicated. (**b**) HoxA9 represses the proximal *CDX4* *cis* element; HoxA10 activates the distal *CDX4* *cis* element. U937 cells were transfected with an artificial promoter/reporter construct with three copies of the −65 to −100 bp *CDX4* sequence (proximal *cis* element) or −100 to −150-bp *CDX4* sequence (distal *cis* element) or empty minimal promoter/reporter control vector (subtracted as background). Cells were co-transfected with vectors to overexpress HoxA9, HoxA10 or both (or control expression vector) and analyzed for reporter activity with or without differentiation. Statistically significant differences in reporter activity are indicated by * and ** for HoxA9 overexpression versus control expression vector; *** for differentiated versus nondifferentiated transfectants overexpressing HoxA9; # and ## for HoxA10 overexpression versus control expression vector; and ### for differentiated versus nondifferentiated in transfectants overexpressing HoxA10. Some differences that are not statistically significant are also indicated. (**c**) Cdx4 influences the proximal *CDX4* *cis* element in a HoxA9-dependent manner. U937 cells were transfected with an artificial promoter/reporter construct with multiple copies of the proximal *CDX4* *cis* element (or control vector) and combinations of vectors to overexpress Cdx4 and knockdown HoxA9 (or control vectors). Reporter assays were performed with or without differentiation. Statistically significant differences in reporter activity are indicated by * and *** for Cdx4 overexpression versus control expression vector and ** and # for HoxA9 knockdown versus control vector in assays with Cdx4 overexpression.

These studies identify a *cis* element between −65 and −100 bp in the *CDX4* promoter that is influenced by HoxA9 (Figure 1b). We introduced mutations into the 150-bp *CDX4* promoter construct to disrupt this *cis* element −86 to −94 bp, or the more distal, HoxA10-binding *cis* element (−139 to −146 bp). The promoter constructs were co-transfected into U937 cells with vectors to overexpress HoxA9, HoxA10 or both. Mutation of the proximal *cis* element blocked repression by overexpressed HoxA9 and increased activation by overexpressed HoxA10 relative to the effect of these proteins on the wild-type 150-bp construct (*P*<0.001, *n*=6; Figure 3a). Reporter expression from the proximal*-cis*-element-mutant construct in HoxA10-overexpressing transfectants was not significantly different with versus without HoxA9 overexpression (Figure 3a). HoxA10 did not activate a 150-bp *CDX4* construct with mutation of the distal *cis* element, but HoxA9 was significantly more efficient at repressing this construct versus the wild-type 150-bp *CDX4* construct (*P*<0.002, *n*=6; Figure 3a). The effect of co-overexpressing HoxA9+HoxA10 on activity of the distal-*cis*-element-mutant construct was not significantly different than HoxA9 alone (Figure 3a).

To eliminate cross-influences between *CDX4* *cis* elements, we generated artificial promoter constructs with three copies of either the −100 to −150 or −65 to −100 bp *CDX4* sequence linked to a minimal promoter and reporter. These constructs (or minimal promoter/reporter control) were co-transfected into U937 cells with vectors to overexpress HoxA9, HoxA10 or both (or control vector). Overexpressed HoxA9 significantly decreased activity of the −65 to −100 bp *CDX4* construct (*P*<0.0001, *n*=6 versus control expression vector) and this effect was greater in differentiated versus undifferentiated transfectants (*P*=0.01, *n*=6; Figure 3b). HoxA10 overexpression did not alter activity of this construct (*P*=0.4, *n*=6 relative to control expression vector) and there was no difference in activity in transfectants with HoxA9 versus HoxA9+HoxA10 (*P*=0.6, *n*=6). The −100 to −150 bp *CDX4* construct was activated by overexpressed HoxA10 (*P*<0.001, *n*=6 versus control expression vector) and this effect was more efficient in undifferentiated transfectants (*P*<0.001, *n*=6; Figure 3b). Overexpression of HoxA9 did not influence the −100 to −150 bp construct (*P*=0.7, *n*=6) and there was no difference in activity in transfectants with HoxA10+HoxA9 versus HoxA10 alone (*P*=0.6, *n*=6).

We hypothesized that the overexpression of Cdx4 would decrease the activity of the proximal *CDX4* *cis* element as HoxA9 represses this *cis* element and Cdx4 activates *HOXA9* transcription. To test this, we co-transfected U937 cells with the −65 to −100 bp *CDX4* reporter construct and vectors to overexpress Cdx4 with or without HoxA9 knockdown. Cdx4 overexpression significantly decreased the activity of the proximal *CDX4* *cis* element (*P*<0.001, *n*=6 versus control expression vector) and this was reversed by HoxA9 knockdown (Figure 3c).

Activity of the empty minimal promoter/reporter vector was not influenced by overexpression of HoxA9, HoxA10 or Cdx4 or differentiation and was subtracted as background.

### HoxA9 interacts with the CDX4 promoter

**Figure 4:**
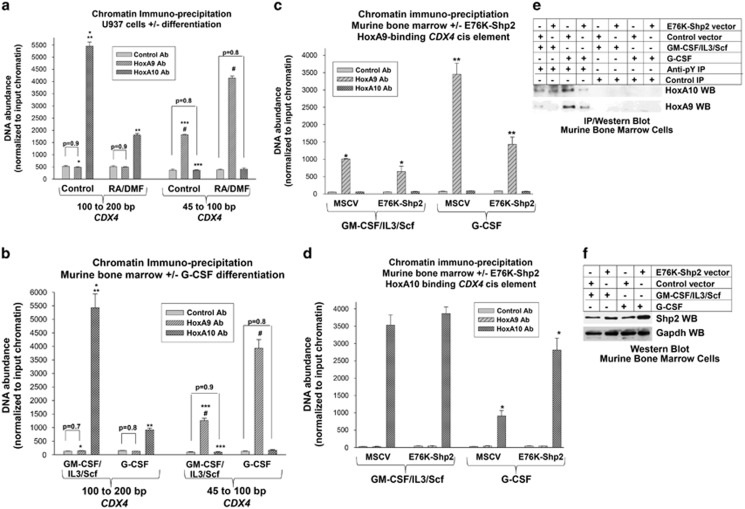
HoxA9 interacts with the proximal *CDX4* promoter *cis* element *in vivo*. (**a**) *In vivo* interaction of HoxA9 with the proximal *CDX4* *cis* element increases during differentiation of U937 cells. U937 cells were analyzed by chromatin co-immunoprecipitation with antibody to HoxA9, HoxA10 or irrelevant control antibody. Precipitating chromatin was amplified by real-time PCR with primers flanking regions of the *CDX4* gene. Cells were analyzed with or without differentiation. Statistically significant differences in co-precipitation of chromatin sequences are indicated by * and *** for HoxA9 antibody versus HoxA10 antibody; ** and # for differentiated versus undifferentiated cells. Differences of *P*<0.01 were considered statistically significant. (**b**) *In vivo* interaction of HoxA9 with the proximal *CDX4* *cis* element increases during *ex vivo* differentiation of murine bone marrow cells. Bone marrow mononuclear cells were harvested from wild-type mice and cultured in GM-CSF, interleukin 3 (IL3) and Scf followed by separation of CD34+ cells (myeloid progenitor conditions) or differentiation with G-CSF. Chromatin was co-precipitated and analyzed as above. Statistically significant differences in co-precipitation of chromatin sequences are indicated by * and *** for HoxA9 antibody versus HoxA10 antibody, ** and # for G-CSF-differentiated cells versus cells cultured under myeloid progenitor conditions. (**c**) Constitutively active Shp2 blocks binding of HoxA9 to the *CDX4* promoter during myeloid differentiation. Bone marrow mononuclear cells were harvested from mice, transduced with a retroviral vector to express E76K-Shp2 (or control vector) and cultured in GM-CSF, IL3 and Scf followed by separation of CD34+ cells (myeloid progenitor conditions) or differentiation with G-CSF. Chromatin was co-precipitated and analyzed by real-time PCR with primers flanking the proximal, HoxA9-binding *CDX4* *cis* element. Statistically significant differences with versus without expression of E76K-Shp2 are indicated by * or **. (**d**) Constitutively active Shp2 increases the binding of HoxA10 to the *CDX4* promoter during myeloid differentiation. Bone marrow cells were transduced and cultured as described above and co-precipitating chromatin was analyzed for binding to the distal, HoxA10-binding *CDX4* *cis* element. Statistically significant differences with versus without expression of E76K-Shp2 are indicated by *. (**e**) Constitutive Shp2 activity decreases tyrosine phosphorylation of HoxA9 and HoxA10 in murine bone marrow myeloid progenitor cells undergoing G-CSF-induced differentiation. Murine bone marrow cells were transduced and treated as described above. Cell lysates were immunoprecipitated with an antibody to phosphotyrosine and immunoprecipitates were analyzed by western blots probed for HoxA9 or HoxA10. Equivalence of protein in the immunoprecipitation samples is addressed in **f**. (**f**) Shp2 expression is increased in cells transduced with E76K-Shp2 expression vector. Lysates from the cells described in **e** above were also analyzed for by western blots probed with antibodies to Shp2 and Gapdh (as a loading control).

We investigated HoxA9 binding to the *CDX4* promoter by chromatin immunoprecipitation. For these studies, chromatin was co-precipitated from U937 cells with an antibody to HoxA9, or HoxA10, or irrelevant antibody. Precipitated chromatin (sheared to <100 bp) was amplified by PCR with primers flanking the proximal (−45 to −100 bp) or distal (−100 to −200 bp) *cis* element. HoxA9 specifically interacted with the proximal *CDX4* *cis* element with a significant increase in interaction on differentiation (*P*<0.001, *n*=3; Figure 4a). HoxA9 did not co-precipitate the distal *cis* element or two irrelevant *CDX4* sequences (from the distal promoter or intron 1; not shown). HoxA10 interacted with the distal *cis* element with a significant decrease in interaction during differentiation (*P*<0.01, *n*=3), but did not interact with the proximal *cis* element (Figure 4a). Cdx4 did not interact with either *CDX4* *cis* element (not shown).

We performed similar chromatin immunoprecipitation using primary murine cells. Bone marrow mononuclear cells were cultured 48 h in granulocyte–macrophage colony-stimulating factor (GM-CSF), interleukin 3 and Scf, and CD34+ cells were isolated (referred to as myeloid progenitor conditions; >70% of these cells are Sca1−ckit+CD34+CD38^−^Gr1^−^). Unlike human bone marrow populations, CD34+ murine bone marrow cells are predominantly committed progenitor populations (and CD38+ cells are differentiating progenitors), not cells with HSC function.^35, 36^ Some cells were differentiated with G-CSF (>80% of these cells are ckit^−^CD34^−^CD38^+^Gr1^+^). We found specific binding of endogenous HoxA9 to the proximal, but not the distal, *CDX4* *cis* element that was significantly greater in G-CSF differentiated cells in comparison with myeloid progenitor cells (*P*<0.0001, *n*=3; Figure 4b). Conversely, the specific binding of endogenous HoxA10 to the distal *CDX4* *cis* element decreased significantly in response to G-CSF (*P*<0.0001, *n*=3; Figure 4b).

To investigate the influence of tyrosine phosphorylation on binding of endogenous HoxA9 and HoxA10 to the *CDX4* promoter, bone marrow mononuclear cells were transduced with a retroviral vector to express constitutively active Shp2 (E76K) or control vector (MSCV). Transduced cells were analyzed by chromatin immunoprecipitation, as above. We found significantly less binding of HoxA9 to the *CDX4* promoter in G-CSF-differentiated cells that were transduced with E76K-Shp2 vector versus MSCV control vector (*P*<0.001, *n*=3; Figure 4c). Conversely, HoxA10 binding to the *CDX4* promoter was significantly greater in G-CSF-differentiated, E76K-Shp2 transduced cells in comparison with control cells (*P*<0.001, *n*=3; Figure 4d).

To confirm the effect of constitutive Shp2 activity on phosphorylation of HoxA9 or HoxA10 during G-CSF-induced differentiation, lysates from the transduced cells were analyzed by phosphotyrosine immunoprecipitation followed by western blot for HoxA9 or HoxA10. Consistent with our prior studies, we found less tyrosine phosphorylation of HoxA9 and HoxA10 in cells transduced with E76K- Shp2 vector versus control vector during G-CSF-induced differentiation (Figure 4e). In control studies with these lysates, we verified increased Shp2 expression in E76K-Shp2 vector-transduced cells and equivalent input protein in the immunoprecipitation experiments (Figure 4f). In control studies, we confirmed that the expression of E76K-Shp2 does not alter expression of endogenous HoxA9 or HoxA10, as we demonstrated in prior studies.^12, 21, 24^

**Figure 5:**
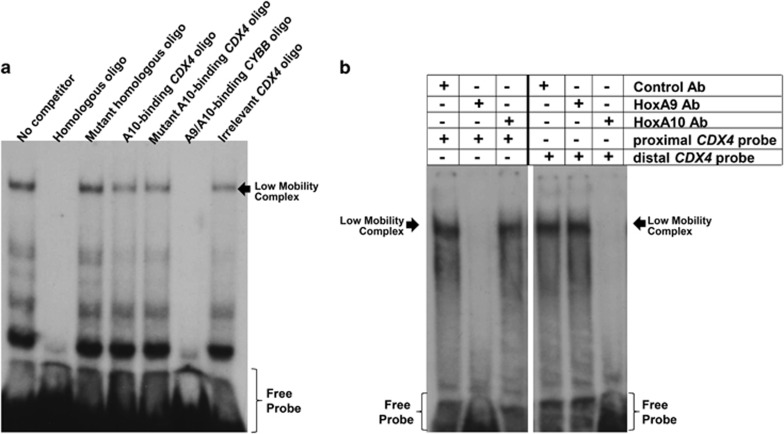
HoxA9 interacts with the proximal *CDX4* *cis* element *in vitro*. (**a**) The proximal *CDX4* *cis* element binds a protein complex *in vitro* that is not cross-reactive with the distal *cis* element. Electrophoretic mobility shift assay (EMSA) was performed with nuclear proteins from U937 cells and a radiolabeled, double-stranded oligonucleotide probe representing the −68 to −104 bp sequence from the *CDX4* promoter with or without unlabeled double-stranded oligonucleotide competitors including homologous *CDX4* sequence (± mutation in the Hox-binding consensus), the −138 to −150-bp HoxA10-binding *CDX4* sequence (± mutation in the Hox-binding consensus), the HoxA9/HoxA10-binding *cis* element from the *CYBB* promoter or an irrelevant oligonucleotide, as indicated. (**b**) HoxA9 binds to the proximal, but not distal, *CDX4* *cis* element. EMSA was performed with U937 nuclear proteins and radiolabeled, double-stranded oligonucleotide probe representing −68 to −104 or −138 to −150-bp *CDX4* promoter sequence with or without an antibody to HoxA9 or HoxA10 or an irrelevant control antibody.

We also investigated HoxA9 interaction with the *CDX4* promoter by electrophoretic mobility shift assays using oligonucleotide probes representing the proximal (−65 to −100 bp) or distal (−100 to −150 bp) *cis* elements and U937 nuclear proteins. Some assays with the proximal *cis* element probe were incubated with unlabeled, oligonucleotides competitors. We found the proximal *cis* element bound a low-mobility complex (Figure 5a), similar to the HoxA10-containing complex that binds the distal *CDX4* cis element (Figure 5b). Protein binding to the proximal *cis* element was disrupted by excess homologous oligonucleotide, but not homologous oligonucleotide with mutation of the Hox-consensus, or distal *cis* element oligonucleotide (Figure 5a). Other assays were incubated with HoxA9, HoxA10 or irrelevant control antibody. Antibody to HoxA9, but not HoxA10, disrupted the proximal *cis* element complex, and antibody to HoxA10, but not HoxA9, disrupted the distal *cis* element complex (Figure 5b).

### HoxA9 decreases expression of Cdx4 in myeloid cells

**Figure 6:**
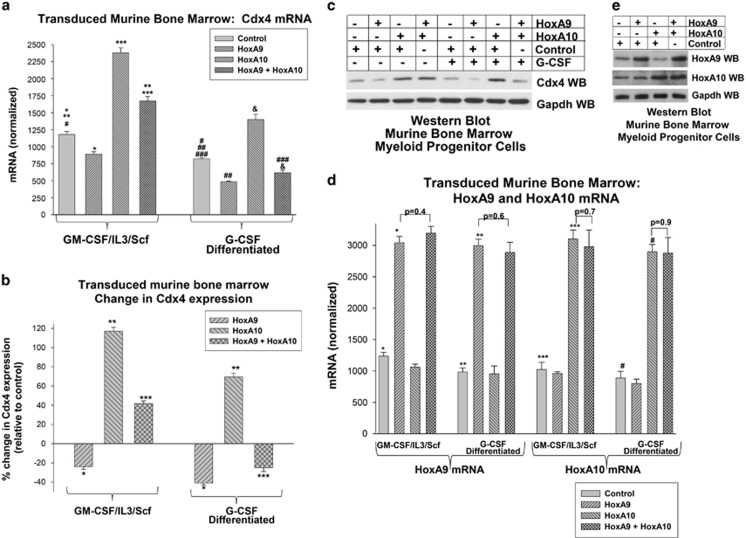
Overexpression of HoxA9 decreases Cdx4 murine myeloid progenitor cells. (**a**) Overexpression of HoxA9 in murine myeloid progenitor cells decreases Cdx4 mRNA and antagonizes HoxA10-induced Cdx4 expression. Murine bone marrow cells were transduced with vectors to overexpress HoxA9, HoxA10, both or control expression vector. Cells were cultured in GM-CSF, interleukin 3 (IL3) and Scf followed by CD34+ cells separation (myeloid progenitor conditions) or differentiation with G-CSF. Cells were analyzed for Cdx4 mRNA by real-time PCR. Statistically significant differences in Cdx4 mRNA abundance are indicated by * and ## for HoxA9 overexpression versus control expression vector; *** and & for HoxA9 overexpression versus control expression vector in cells co-overexpressing HoxA10; ** or ### for HoxA9+HoxA10 overexpression versus control expression vector; and # for GMP versus G-CSF-differentiation. Differences of *P*<0.01 are considered statistically significant. (**b**) Differentiation with G-CSF switches the effect of co-overexpressing HoxA9+HoxA10 from increasing to impairing Cdx4 expression. Data from the experiments above were analyzed as % change in Cdx4 expression in HoxA9, HoxA10 or HoxA9+HoA10 overexpressing cells versus control vector-transduced cells. Statistically significant differences in Cdx4 expression in myeloid progenitor cells versus G-CSF differentiated cells are indicated by *, ** or ***. (**c**) HoxA9 overexpression decreases Cdx4 protein in primary murine bone marrow cells. Lysate proteins from the transduced murine bone marrow cells, described above, were analyzed for protein expression. Western blots were serially probed with antibodies to Cdx4 or Gapdh (loading control). (**d**) HoxA9 and HoxA10 are equivalently overexpressed in transduced primary murine bone marrow cells. The transduced bone marrow cells, describe above, were analyzed for HoxA9 and HoxA10 mRNA by real-time PCR. Statistically significant differences in mRNA are indicated by * and ** for HoxA9 overexpression versus control expression vector; *** and # for HoxA10 overexpression versus control expression vector. Some nonstatistically significant differences are also indicated. (**e**) Expression of HoxA9 and HoxA10 protein correlates with mRNA expression in the transduced cells. Western blots of cell lysates from these transduced murine bone marrow cells were analyzed for expression of HoxA9 and HoxA10 protein. Western blots were serially probed with antibodies to HoxA9, HoxA10 and Gapdh (loading control).

We investigated the impact of HoxA9 on endogenous Cdx4 expression using murine bone marrow cells that were transduced with vectors to express HoxA9, HoxA10 or HoxA9+HoxA10 (or empty vector). Cells were cultured under myeloid progenitor conditions or differentiated with G-CSF (see above) and analyzed for Cdx4 mRNA. We found significantly less Cdx4 mRNA in HoxA9-overexpressing cells versus cells transduced with control vector (*P*<0.01 for three independent experiments, analyzed in duplicate; Figure 6a). The HoxA9-induced difference in Cdx4 expression was significantly greater in G-CSF-treated cells versus myeloid progenitors (*P*<0.001, *n*=3; Figure 6b). Conversely, the effect of HoxA10 overexpression on Cdx4 mRNA expression was significantly greater in myeloid progenitor cells versus G-CSF-treated cells (*P*<0.001, *n*=3; Figure 6b). Cdx4 mRNA was significantly greater in HoxA9+HoxA10-overexpressing myeloid progenitors versus control vector-transduced cells (*P*<0.01, *n*=3; Figure 6a). However, Cdx4 mRNA was significantly less in HoxA9+HoxA10-overexpressing, G-CSF treated cells in comparison with similarly treated control vector-transduced cells (*P*<0.01, *n*=3; Figure 6a). Therefore, the effect of co-overexpressing HoxA9+HoxA10 switched from increasing Cdx4 mRNA in comparison with control vector in myeloid progenitor cells to decreased Cdx4 mRNA in comparison with control in G-CSF-differentiated cells (*P*<0.001, *n*=3; Figure 6b).

In these studies, Cdx4 mRNA expression correlated with Cdx4 protein expression (Figure 6c). HoxA9 and HoxA10 mRNA (Figure 6d) and protein (Figure 6e) were equivalently overexpressed.

### Mll-Ell influences CDX4 transcription in a HoxA9/HoxA10-dependent manner

**Figure 7:**
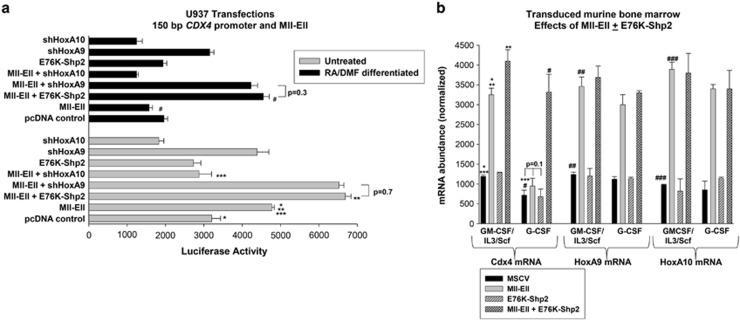
Mll-Ell influences Cdx4 expression in a HoxA9/HoxA10-dependent manner. (**a**) Mll-Ell increases *CDX4* promoter activity in myeloid progenitors, but not differentiating myeloid cells; the effect of differentiation is blocked by HoxA9-knockdown or constitutive Shp2 activation. U937 cells were co-transfected with a reporter construct with 150 bp of *CDX4* promoter and vectors to express Mll- Ell with or without vectors to express specific shRNAs for HoxA9 or HoxA10 or E76K-Shp2. Reporter gene assays were performed with or without differentiation. Statistically significant differences in reporter activity are indicated by * for Mll-Ell versus control expression vector; ** and # for expression of E76K-Shp2 versus control expression vector in Ml-Ell expressing transfectants; and *** for expression of HoxA10-specific shRNA versus control shRNA in Mll-Ell expressing transfectants. (**b**) Mll-Ell increases Cdx4 expression in murine myeloid progenitor cells, but not G-CSF-treated cells, and this effect of G-CSF is blocked by constitutive Shp2 activation. Primary murine bone marrow cells were transduced with a vector to express Mll-Ell, E76K-Shp2, Mll-Ell+E76K-Shp2 or control expression vector. Cells were cultured in GM-CSF, interleukin 3 (IL3) and Scf followed by either separation of CD34+ cells (myeloid progenitor conditions) or differentiation with G-CSF, and analyzed for Cdx4, HoxA9, and HoxA10 mRNA by real-time PCR. Statistically significant differences in expression of these genes are indicated by *, ## and ### for with versus without Mll-Ell; ** or # for with versus without E76K-Shp2 in Mll-Ell expressing cells; and *** for myeloid progenitor versus G-CSF-differentiated cells.

As Mll-fusion proteins increase the expressions of both HoxA9 and HoxA10, the influence of such proteins on *CDX4* transcription might be similar to co-overexpressing HoxA9+HoxA10. To investigate this, we co- transfected U937 cells with the 150-bp *CDX4* reporter construct and a vector to express Mll-Ell or empty expression vector, with or without vectors to knockdown HoxA9 or HoxA10. We found that Mll-Ell significantly increased the activity of the *CDX4* promoter in untreated transfectants (*P*<0.001, *n*=3 versus control expression vector), but slightly repressed promoter activity in transfectants undergoing differentiation (Figure 7a). HoxA10 knockdown significantly decreased *CDX4* promoter activation by Mll-Ell in undifferentiated transfectants (*P*<0.001, *n*=3 for Mll-Ell versus Mll-Ell+HoxA10 shRNA) and HoxA9 knockdown significantly increased activation by Mll-Ell with or without differentiation (*P*<0.0001, *n*=3 for Mll-Ell versus Mll-Ell+HoxA9 shRNA; Figure 7a). As inhibiting phosphorylation of HoxA9 and HoxA10 increases *CDX4* promoter activity, we co-transfected U937 cells with the 150-bp *CDX4* promoter construct and vectors to express Mll-Ell plus E76K-Shp2. We found that E76K-Shp2 significantly increased Mll-Ell-induced *CDX4* promoter activation in undifferentiated and differentiating transfectants (*P*<0.001, *n*=3 for Mll-Ell versus Mll-Ell+E76K-Shp2; Figure 7a).

We also investigated the effect of Mll-Ell on the expression of endogenous Cdx4 in murine bone marrow. Bone marrow mononuclear cells were transduced with retroviral vectors to express Mll-Ell, E76K-Shp2, both or control vector and cultured under myeloid progenitor or G-CSF-differentiation conditions (defined above). We found significantly more Cdx4 mRNA in Mll-Ell-transduced versus control vector-transduced myeloid progenitor cells (*P*<0.001, *n*=3; Figure 7b). This effect of Mll-Ell on Cdx4 mRNA was abolished by G-CSF differentiation (Figure 7b). In cells co-expressing Mll-Ell+E76K-Shp2, Cdx4 mRNA was significantly greater than in control vector-transduced cells under myeloid progenitor and G-CSF differentiation conditions (*P*<0.01, *n*=3; Figure 7b). HoxA9 and HoxA10 mRNA were both increased by Mll-Ell expression (*P*<0.0001, *n*=3 versus control expression vector; Figure 7b). Mll-Ell expression was verified by real-time PCR with primers spanning the fusion (not shown).

### Shp2 inhibition decreases Cdx4 expression in Hox-overexpressing AML

**Figure 8:**
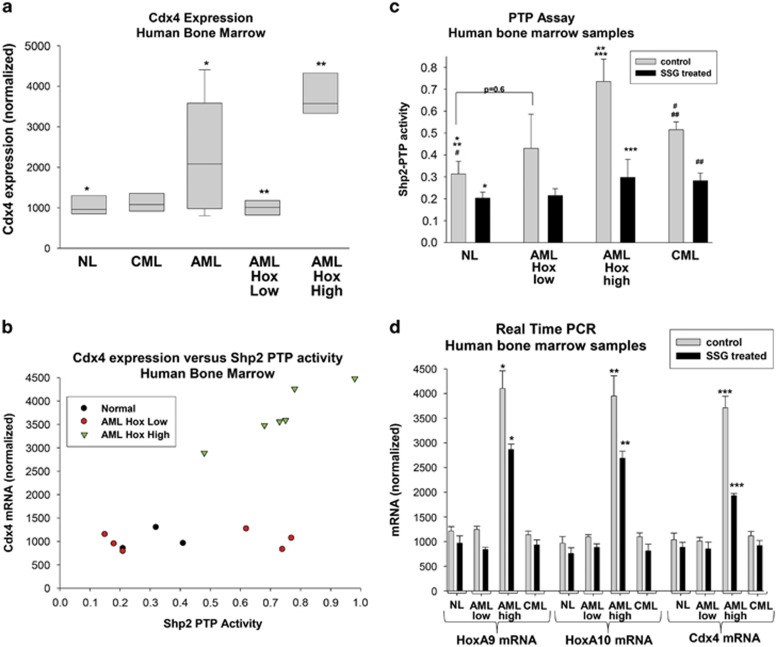
Shp2 activity influences Cdx4 expression in Hox-overexpressing human AML. (**a**) Increased Cdx4 expression correlates with increased expression of HoxA9 and HoxA10 in AML. Human bone marrow CD34+ cells from subjects with chronic myeloid leukemia (CML), AML or non-leukemic control subjects (NL) were analyzed by real-time PCR for expression of HoxA9, HoxA10 or Cdx4 mRNA. AML samples were separated into groups with increased HoxA9/HoxA10 expression (AML Hox high) or without increased Hox expression (AML Hox low). Statistically significant differences are indicated by * or **. (**b**) Cdx4 expression correlates with Shp2 activity in AML. Cells in the AML Hox-high and AML Hox-low groups (and control cells) above were analyzed for Shp2 activity by functional protein tyrosine phosphatase assay. Results were graphed as Shp2 activity versus Cdx4 mRNA. (**c**) Treatment with SSG decreases Shp2 activity in human bone marrow cells. The cells described above were also assayed for Shp2 protein tyrosine phosphatase activity with or without treatment with SSG. Statistically significant differences with versus without SSG are indicated by *, *** and ##. Statistically significant difference between control and leukemia samples are indicated by ** and #. (**d**) Treatment with SSG decreases the expressions of Cdx4, HoxA9 and HoxA10 in AML cells. The cells described above were also analyzed by real-time PCR for the expressions of Cdx4, HoxA9 and HoxA10 mRNAs. Statistically significant differences with versus without SSG in Hox-high AML are indicated by *, ** or ***.

We also investigated the impact of HoxA9 and HoxA10 expression and Shp2 activity on Cdx4 expression in human AML. We analyzed CD34^+^ bone marrow cells from subjects with AML (16 total), chronic myeloid leukemia (5 total) or normal control individuals (4 total). Cells were assayed for HoxA9, HoxA10 and Cdx4 mRNA. AML samples were grouped for analysis as high Hox-expressing (⩾2 s.d. above the control mean; six samples) or low Hox-expressing (<2 s.d. above control; six samples). None of these samples were known to harbor translocations or duplications involving the *MLL1* gene, but the Hox-high group included three samples that evolved from a myelodysplasia and two with relapsed/refractory disease. The Hox-low samples were all *de novo* AML. We found that Cdx4 mRNA expression was significantly greater in AML bone marrow cells relative to bone marrow from either chronic myeloid leukemia or control subjects (*P*<0.01 by three-way analysis of variance; Figure 8a). Cdx4 mRNA was significantly greater in the Hox-high AML group versus Hox-low AML (*P*<0.0001, *n*=3; Figure 8a).

We also analyzed the Shp2 phosphatase activity in some samples. Linear regression analysis identified a positive correlation between Shp2-protein tyrosine phosphatase activity and Cdx4 mRNA in the bone marrow of Hox-high AML (*P*=0.01, *n*=6), but not Hox-Low AML (*P*>0.6, *n*=6) or control bone marrow (*P*>0.4, *n*=4; Figure 8b). We treated a subset of samples with sodium stibogluconate (SSG), a specific inhibitor of Shp1/Shp2.^36^ Although these samples were not characterized for Shp2 mutations, we previously determined that SSG inhibits both wild-type and constitutively active Shp2 (not shown). We found that SSG treatment significantly decreased Shp2-PTP activity in all of the samples (*P*<0.01; Figure 8c). Cdx4 expression decreased significantly in the high-Hox-expressing group on treatment with SSG (*P*<0.0001, *n*=4), as did the expressions of HoxA9 and HoxA10 (*P*<0.01, *n*=4; Figure 8d). Effects of Shp2 inhibition on expression of HoxA9, HoxA10 and Cdx4 mRNA in the other groups of samples did not reach statistical significance.

## Discussion

**Figure 9:**
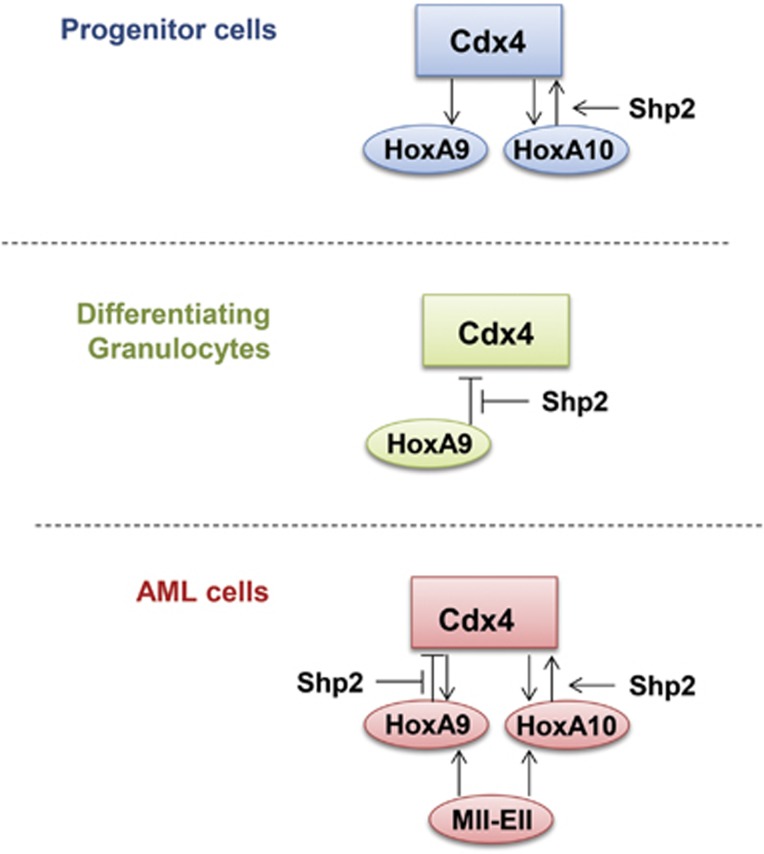
Graphical representation of the influence of HoxA9 and HoxA10 on Cdx4 expression. Influences in myeloid progenitor cells are compared with differentiating granulocytes and AML cells.

In these studies, we identify a mechanism that decreases *CDX4* transcription during myelopoiesis. As Cdx4 activates transcription of genes involved in expanding hematopoietic stem and progenitor cells, decreased expression of Cdx4 contributes to proliferation arrest as myelopoiesis proceeds. We find that HoxA9 breaks the positive feedback loop between Cdx4 and HoxA10 in a differentiation stage-specific manner. Therefore, HoxA10 and Cdx4 activate each other's promoters in myeloid progenitor cells, leading to cytokine hypersensitivity and progenitor expansion via HoxA10 target genes such as FGF2, TGFB2 and ITGB3.^19, 34, 37^ In contrast, HoxA9 represses the *CDX4* promoter in differentiating myeloid cells; inhibiting Cdx4-dependent events. Our current studies also determine that this mechanism is circumvented by constitutive activation of Shp2 in Hox-overexpressing forms of leukemia (Figure 9). This results in the sustained activation of *CDX4* transcription by nonphosphorylated HoxA10 and impaired *CDX4* repression by nonphosphorylated HoxA9 in cells with dysregulated Shp2 activity.

A subset of poor prognosis AML is characterized by aberrant expression of a group of HD proteins, including HoxB3, B4, A7–11, Cdx2, Cdx4 and Meis1.^3, 4, 5, 38^ This includes AML with *MLL1* translocation or partial tandem duplication, but it also a poor prognosis subset with normal cytogenetics.^3, 4, 38^ We found that co-overexpression of HoxA9+HoxA10, or expression of a leukemia-associated Mll-fusion protein (Mll-Ell), facilitates HoxA10-dependent *CDX4* transcription in myeloid progenitor cells. However, Cdx4 expression decreases in cells exposed to differentiating cytokines. We found that this balance was shifted and Cdx4 expression was sustained by maneuvers that impair cytokine-induced phosphorylation of HoxA9 and HoxA10, including constitutive activation of Shp2. Therefore, sustained, aberrant *CDX4* transcription in 11q23-AML requires a secondary, Shp2-activating mutation. Consistent with this, we found that inhibition of Shp2 in human AML samples with increased HoxA9, HoxA10 and Cdx4 decreased the expression of these HD proteins.

In previous studies, we found that HoxA10 represses *CYBB* and *NCF2* transcription in myeloid progenitor cells by interacting with tandem *cis* elements in these genes, but HoxA9 activates the same *cis* elements during phagocyte differentiation.^20, 21, 22, 23, 24^ We also identified *FGF2* as a target gene for HoxA9 and HoxA10, but *FGF2* is activated by cooperative interaction of the two Hox proteins with a common *cis* element throughout myelopoiesis.^28^ Mll-Ell increases *FGF2* transcription and autocrine production of Fgf2 in myeloid cells, and tyrosine phosphorylation of HoxA9 and HoxA10 does not influence this activity.^25^ Fgf2 expands bone marrow progenitor cells and participates in phagocyte effector functions.^25, 34, 39^ In contrast, *CDX4* represents the first target gene regulated by the interaction of HoxA9 and HoxA10 with different *cis* elements. The mode of *CDX4* regulation by HoxA9 and HoxA10 is also previously undescribed; transcription is activated by HoxA10 in myeloid progenitors and repressed by HoxA9 in differentiating cells. In all three genes with antagonistic regulation by HoxA9 and HoxA10 (*CYBB, NCF2, CDX4*), phosphorylation of conserved tyrosine residues in the DNA-binding HD decreases the binding of HoxA10, but increases HoxA9 binding.^20, 21, 22, 23, 24^

Our studies identify three classes of HoxA9/HoxA10 target genes. The first are the genes involved in phagocyte effector functions that are repressed by HoxA10 in progenitors, but activated by HoxA9 during myelopoiesis. The second are the genes involved in myeloid progenitor expansion that are activated by HoxA10 in progenitors, but repressed by HoxA9 during myelopoiesis. The third are the genes involved in both phagocyte function and progenitor expansion that are activated by cooperation between HoxA9 and HoxA10 throughout myelopoiesis. Ongoing studies in our laboratory will determine if these hypothetical categories are substantiated.

The mechanism for aberrant *CDX4* transcription described in these studies is clinically relevant as activating mutations of the Shp2 gene are found 11q23-AML.^27^ Shp2 is also activated by leukemia-associated, activating Flt3 mutations.^40^ Such mutations occur frequently in AML and are enriched in the Hox-overexpressing subset.^27, 41^ Consistent with this, gene expression profiling studies correlate increased Cdx4 expression with *MLL1* gene translocations, Flt3 mutations and progression from MDS to AML, but not with progression from chronic phase to blast crisis in chronic myeloid leukemia.^42, 43, 44^ Although our sample size is small, we also found correlation between Hox expression, Cdx4 expression and Shp2-PTP activity in a subset of AML. Determining the overall incidence of Hox overexpression and altered expression of target genes, such as Cdx4, will require larger studies. The incidence of Hox overexpression in our small cohort may be slightly skewed by the overrepresentation of patients with advanced and refractory disease in our referral center cohort.

Understanding the cooperation between these leukemia-associated mutations will be of interest for identifying potential, specific therapeutic targets for this treatment refractory form of AML. Our studies suggest Shp2 would be one targetable candidate.

## Materials and methods

### Plasmids

HoxA10 cDNA was obtained from C Largman (University of California, San Francisco).^45^ Mll-Ell cDNA was obtained from DE Zhang (University of California, San Diego). Cdx4 and HoxA9 cDNAs were obtained by PCR from U937 cells. HoxA9, HoxA10, Cdx4 or Mll-Ell cDNAs were subcloned into pcDNAamp (for transfections) and MSCV (for retrovirus production; Stratagene, La Jolla, CA, USA).^46^ Tyrosine mutant HoxA9 (Y212F/Y225F-HoxA9) or HoxA10 (Y326F/Y343F-HoxA10) was generated by site-directed mutagenesis, as described.^21, 23^ Shp2 and E76K-Shp2 plasmids have been previously described.^21, 23^ HoxA9- or HoxA10-specific shRNAs were designed with the Promega website (Madison, WI, USA). Double-stranded oligonucleotides with complementary sequences separated by a hairpin were subcloned into pLKO.1puro vector (from K Rundell, Northwestern University, Chicago). Several sequences were tested and the most efficient combined. Matched shRNAs with scrambled sequences were controls.

The *CDX4* 5′ flank was amplified by PCR from U937 chromatin, sequenced to ensure identity with the ENSEMBL data base and subcloned into pGL3-E vector (Promega).^19^ Constructs with three copies of −139 to −150 or −68 to −104 bp of the *CDX4* promoter were generated in the pGL3- promoter vector.

### Oligonucleotides

Oligonucleotides were synthesized by MWG Biotech (Piedmont, NC, USA). For electrophoretic mobility shift assay :-139 to −150 bp of *CDX4* (5′-GTGGGATGATGTAGCCTGAGGG-3′, mutant 5′-GTGGGATG**GCT**TAGCCTGAGGG-3′) or −68 to −104 bp of *CDX4* (5′-ACAACTACGTACTGATAAGTTTATTCTCTGCTGCTT-3′, mutant (5′-ACAACTACGTACTGATAAG**CAC**ATTCTCTGCTGCTT-3′). For PCR for chromatin immunoprecipitation (5′–3′): *CDX4* (HoxA10-binding site) (F-TATGTAAAAGCCTGAAGCCCCTT, R-AAGCTCTTTTGCACCCCTC), *CDX4* (HoxA9-binding site) (F-TGCAAAAGAGCTTGCGGCACAACTAC, R-TAAGCCATCCTGAAGTCCCTGTAA). Real-time PCR primers for mRNA expression were previously described.^19, 28, 34^

### Myeloid cell line transfections and assays

The human myeloid cell line U937^29^ was obtained from Andrew Kraft (Hollings Cancer Center, University of South Carolina, Charleston). Cells were maintained as described (as per manufacturer's instructions: Stratagene). U937 cells were co-transfected with a luciferase reporter vector (pGL3-E) containing 1.4 kb, 450, 150, 100 or 65 bp of *CDX4* 5′ flank sequences, or pGL3-E control (30 pg) and a HoxA9 expression vector or control (50 pg). The 150-bp construct was co-transfected with combinations of vectors (50 pg) to overexpress or knockdown HoxA9 and HoxA10; vectors to overexpress HoxA9 or HoxA10 with HD-Y residues changed to F (HD-Y-mutant); vectors to co-overexpress combinations of Wt or HD-Y mutant HoxA9 or HoxA10+constitutively active Shp2 (E76K); or vectors to express Mll-Ell+E76K-Shp2. Other cells were co-transfected with a minimal promoter-luciferase reporter vector with three copies of the distal (−139 to −146 bp) or proximal (−84 to −96 bp) *CDX4* *cis* element and combinations of vectors to overexpress HoxA9, HoxA10 or Cdx4 (or control vector)+vectors to express specific shRNAs for HoxA9 or HoxA10 (or scrambled control; 50 pg). Cells were transfected with a E-galactosidase reporter to control for transfection efficiency.

### Murine bone marrow studies

Animal studies were performed according to the ACUC-approved protocols at Northwestern University and Jesse Brown VA.

Bone marrow mononuclear cells were obtained from femurs of C57/BL6 mice and cultured for 24 h in DME with 10% fetal calf serum, 1% penicillin–streptomycin, 20 ng/ml murine GM-CSF (R&D Systems Inc., Minneapolis, MN, USA), 20 ng/ml murine recombinant IL3 (R&D Systems) and 100 ng/ml murine recombinant stem cell factor (Scf; R&D Systems; 2 × 105 cells/ml). Cells were maintained 48 h in GM-CSF+IL3+Scf followed by isolation of CD34+ cells (using the Miltenyi magnetic bead system, Miltenyi Biotechnology, Auburn, CA, USA) or differentiation with 20 ng/ml of G-CSF.^12^

Retrovirus (~107 PFU/ml) was generated with HoxA10/MSCV, HoxA9/MSCV, E76K-Shp2/MSCV or control MSCV using the Phoenix packaging line according to manufacturer's instructions (Stratagene). Bone marrow mononuclear cells were cultured 24 h in 20 ng/ml IL3, 20 ng/ml GM-CSF and 100 ng/ml SCF, incubated with retroviral supernatant supplemented with polybrene (6 pg/ml) and cultured 48 h in GM-CSF, IL3 and Scf±G-CSF.^12^ Transduced cells were selected in the appropriate antibiotic (G418 or puromycin with the various vectors) post transduction. We find that 70–75% if the cells are consistently transduced (preselection) with this technique.

### Human leukemia cells

Human studies were performed with the approval of the Northwestern University institutional review board. Bone marrow was obtained from leukemia subjects at the time of diagnostic evaluation or from individuals without leukemia. CD34+ cells were isolated (using the Miltenyi magnetic bead system) and cultured for 24 h in human GM-CSF, IL3 and Scf (at concentrations indicated above).

### Quantitative real-time PCR

RNA was isolated using Trizol (Gibco-BRL, Gaithersburg MD, USA) and tested for integrity by denaturing electrophoresis. Primers were designed with Applied Biosystems software and real-time PCR performed using the SYBR green ‘standard curve' method. Results were normalized to 18S and actin (mRNA) or input chromatin (co-precipitated chromatin).

### Chromatin immunoprecipitation

U937 cells were briefly treated with formaldehyde to generate DNA–protein cross links. Cell lysates were sonicated to generate chromatin fragments of <100 bp and immunoprecipitated with antibody to HoxA9, HoxA10 or Cdx4 or control antibody (Abcam, Cambridge, MA, USA).^28, 34, 47^ Precipitated chromatin was analyzed by PCR.

### Western blots and phosphatase assays

Cells were lysed by boiling in 2 × SDS sample buffer, proteins (50 pg), separated by SDS–polyacrylamide gel electrophoresis, transferred to nitrocellulose and western blots serially probed with antibodies to HoxA9, HoxA10, Cdx4 and GAPDH (loading control). Each experiment was repeated three times with different lysates and a representative blot is shown. Immunoprecipitation studies were performed with anti-phosphotyrosine antibody (clone 4G10, Millipore, Billerica, MA, USA) under nondenaturing conditions.^12, 23, 24^ Immunoprecipitates were separated by SDS–polyacrylamide gel electrophoresis and western blots probed with antibodies to HoxA9 or HoxA10. Nonimmunoprecipitated lysates were used for control western blots that were probed with Shp2 and GAPDH antibodies. For phosphatase assays, cells were lysed in RIPA buffer and immunoprecipitated with Shp2 or irrelevant control antibody and assayed for functional PTP activity as previously described.^12, 23, 24^

### Electrophoretic mobility shift assays

Nuclear proteins were extracted by Dignam's method.^48, 49^ Oligonucleotides probes were prepared and electrophoretic mobility shift assay was performed as described.^23, 47, 48^ HoxA9, HoxA10 or irrelevant control antibody was added to some assays. Competition studies were performed with double-stranded oligonucleotides at 200-fold molar excess.^49, 50^ At least three batches of nuclear proteins were tested in two independent experiments. Protein integrity and loading was determined in electrophoretic mobility shift assay with a CCAAT box probe.

### Genomic sequence analysis

Conserved sequences and Hox-consensus sequences were identified using VISTA (Genomics Division of the Lawrence Berkley National Laboratory, Berkley, CA, USA^51, 52, 53, 54^).

### Statistical analysis

Statistical significance was determined by Student's *t*-test and analysis of variance using SigmaPlot software (SigmaStat, Chicago, IL, USA). Error bars represent s.e.
